# Supplementary material for: Green Extraction of Graphene from Natural Mineral Shungite
Source: Nanomaterials (Basel). 2022 Dec 7;12(24):4356. doi: 10.3390/nano12244356 (PMC9787502; doi:10.3390/nano12244356)
Supplement: Supplementary file 1 [file nanomaterials-12-04356-s001.zip › nanomaterials-2054477-supplementary.pdf]

nanomaterials

Article

Green-Extraction of Graphene from Natural Mineral Shungite - Supplementary Material

Anastasia Novikova and Alina Karabchevsky *

School of Electrical and Computer Engineering, Ben-Gurion University of the Negev, Beer-Sheva 8410501, Israel
* Correspondence: alinak@bgu.ac.il

X-ray photoelectron spectrometry has also been used to quantify the relative intensity of components associated with C 1s and O 1s. The data is presented in Table S1.

Table S1. Elements Identifier and Quantitative assessment of shungite samples before sonication

Name	Peak (BE)	FWHM (eV)	Area (CPS·eV)	Atomic (%)
C1S	284.76	1.16	61792.24	88.62
O1S	532.29	3.60	21751.34	11.38

Table S1 shows the XPS spectra of shungite after deconvolution. The main Shungite peak is carbon at 284.76 eV corresponding to sp2-hybridized carbon (C=C) and the oxygen peak at 532.29 eV corresponds to C=O. Shungite samples before treatment contain 88.62% hybridized carbon, and oxygen 11.38 %, most likely due to the presence of water in the samples.

Next, the samples were processed by the sonication method. The data obtained are presented in Table S2.

Table S2. Elements Identifier and Quantitative assessment of shungite samples after sonication

Name	Peak (BE)	FWHM (eV)	Area (CPS·eV)	Atomic (%)
C1S	284.58	1.46	16150.69	92.41
O1S	533.29	3.28	34194.57	7.59

Table S2 shows the XPS spectra after deconvolution of the sonicated shungite sample. Table S2 shows the peak of the sp2 form of carbon, located at 284.5 eV, which is attributed to carbon. The peak is broadened compared to the peaks before treatment, and the concentration increases to 92.41%. The oxygen peak is shifted by 533.29, which corresponds to C-OH bonds and is 7.59%. The data supports our hypothesis that C=O bonds are broken.

check for updates

Citation: Novikova, A.; Karabchevsky, A. Green-Extraction of Graphene from Natural Mineral Shungite. *Nanomaterials* **2022**, *12*, 4356. https://doi.org/10.3390/nano12244356

Academic Editors: Wugang Liao and Lin Wang

Received: 17 July 2022
Accepted: 22 August 2022
Published: 7 December 2022

Publisher's Note: MDPI stays neutral with regard to jurisdictional claims in published maps and institutional affiliations.

Copyright: © 2022 by the authors. Licensee MDPI, Basel, Switzerland. This article is an open access article distributed under the terms and conditions of the Creative Commons Attribution (CC BY) license (https://creativecommons.org/licenses/by/4.0/).
